# Toward a Sociotechnical Ecosystem for Ethical Screening and Promotion of Mental Health and Well-Being

**DOI:** 10.2196/64790

**Published:** 2025-08-14

**Authors:** Federico Colecchia, Gabriella Spinelli, Dominik Havsteen-Franklin, Monomita Nandy

**Affiliations:** 1 College of Engineering, Design and Physical Sciences, Brunel University of London, Kingston Lane, Uxbridge, UB8 3PH, United Kingdom, 44 018952 ext 66236; 2 College of Arts, Law, and Social Sciences, Brunel University of London, Uxbridge, United Kingdom; 3 Brunel Business School, Brunel University of London, Uxbridge, United Kingdom

**Keywords:** mental health, mental well-being, health screening, digital technologies, predictive analytics, portable electronic devices.

## Abstract

This concept paper delineates the design of a sociotechnical ecosystem for ethical screening and promotion of mental health and well-being, building on the convergence of digital technologies with modern human-centered design methods. Access to individuals’ health and well-being data will enable the generation of actionable insights with different degrees of granularity, for the benefit of individuals, care providers, and business organizations. Critical to the success of the ecosystem are the proactive involvement of all stakeholders, the definition of incentives to encourage engagement, and the promotion of consistent narratives as public institutional messages. The article posits working hypotheses, including the idea that creative externalization of health and well-being data, augmented by advanced physico-digital interactivity, can sustain positive psychological and behavioral change. The theoretical underpinning consists of the integration of existing frameworks across well-being, behavior change, and sustainable business. The article defines a research agenda for expanding socially inclusive dialogue on data governance and policy implications.

## Introduction

Innovation for mental health and well-being has attracted significant attention in recent years due to its potential for promoting the development and growth of individuals, organizations, local communities, and society. It has been estimated that workplace interventions toward improved employee mental well-being in the United Kingdom, including the implementation of screening initiatives and care management programs for those at higher risk of developing more severe conditions, can result in significant net profits for employers. Promotion of mental well-being at work, including risk assessment and stratification, combined with the provision of personalized information, seminars, and workshops for employees, has been estimated to lead to a net profit of over £340,000 (US $460,021.70) per year in savings for a typical business with 500 employees under the assumption that two-thirds of the staff engage in the interventions (GBP to US$ conversion rate at the time of writing was 1.36). Such a profit is driven by reduced costs associated with the assessment and treatment of mental health conditions, lower rates of absence from work, and a reduction in lost productivity due to suboptimal workforce performance [1,2]. The negative societal and economic impact of recent global events such as the 2007‐2008 financial crisis and the COVID-19 pandemic has further highlighted the importance of achieving higher degrees of individual and collective resilience within social and productive systems.

In addition to the relevance of employee mental well-being to business operation and productivity, the increasing prevalence of anxiety, depression, and—more generally—mental ill health has often highlighted infrastructural and capacity limitations of support and care services [3]. Opportunities have been identified for digital technologies to augment existing support and care workflows, for example, to increase efficiency and improve access to mental health services in primary care settings [4]. However, the potential of digitally enhanced systems for streamlining workforce well-being service access at scale—beyond basic functionalities provided by common self-assessment and self-management mobile software applications—is yet to be fulfilled. Recent research has highlighted opportunities for the development of innovative solutions, enabled by modern technologies, to enhance employee well-being [5-8]. Separate studies have considered opportunities for mobile health innovators to contribute to the development of digital mental health ecosystems [9]. However, despite the useful insights generated, these independent lines of research have failed to converge and delineate the design of a sociotechnical ecosystem complex enough to simultaneously address challenges faced by care service providers, employers, and individuals.

The reasons for this lack of convergence are more user-centered than technological in nature and are often associated with a lack of involvement of designers and design researchers together with end users (public and care professionals) in the design and development of digital solutions [10]. Whereas end user involvement has been documented, little information has been provided regarding the nature and scope of the engagement throughout the development process. For this reason, there is often little clarity regarding the benefits associated with designers and health professionals taking the lead in scaffolding and facilitating the generation of innovative design concepts with significant end user and stakeholder involvement. This issue is frequently compounded by preconceptions around mental health conditions and by the associated social stigma, which can further reduce the engagement of the public with support services and can therefore limit the benefits associated with the provision of early-stage mental well-being support [11]. This combination of factors can result in a significant missed opportunity with reference to individuals who, if their signs of psychological distress could be detected and flagged at an earlier stage, could receive additional support before more debilitating symptoms manifest themselves [12].

It can be argued that challenges for care providers and business organizations associated with mental ill health can be conceptualized within the same human-centered systems design framework. In recognition of the relevance of system-level interdependencies within organizations and communities, recent research has targeted emotional, physical, and psycho-social dimensions of health and well-being. A need to consider multi-scale interdependencies within large-scale social systems when designing for innovation toward individual and community well-being is illustrated, among others, by research on agent-based computational modeling of community mental health care processes and population well-being. Such research aims to improve care and support service quality and to reduce the financial burden on taxpayers [13]. The adoption of system-level perspectives has been advocated for the evaluation of public health interventions [14], and interdependences between business and society are well documented [15]. Nonetheless, based on the current literature, it can be argued that no unified framework has, so far, been presented for addressing the needs and requirements of different stakeholders including the public, care providers, and business organizations. A unified approach toward integrating portable electronic devices and modern predictive analytics capabilities within a sociotechnical ecosystem to enable ethical monitoring and promotion of mental health and well-being on a large scale is lacking. On the other hand, ongoing research and development have contributed to the establishment of a mature knowledge base to provide the foundation of such an ecosystem; this is particularly notable with reference to research on emerging as well as more established technologies for monitoring and promoting individual and community well-being in domestic, workplace, and broader social settings [16-19].

This article outlines the theoretical and methodological underpinnings of an ecosystem comprising individuals, care providers, business organizations, and government authorities, aimed at large-scale ethical monitoring and promotion of mental health and well-being. Central to the ecosystem is a commitment to ethical practices in gathering and managing mental health and well-being data across communities, including self-reports and data collected via continuous passive monitoring of individuals’ bio-signals, while rigorously adhering to privacy regulations and achieving clarity in relation to data ownership. The ecosystem, to be designed, implemented, and evaluated in coordination with all stakeholders, will streamline information exchange via a digital platform envisaged for collection, integrity assessment, integration, storage, and analysis of health and well-being data. It is expected that the ecosystem will contribute to more efficient and equitable access of individuals to mental health support and care services by further increasing the efficiency of existing triaging processes, among others. In the first instance, the ecosystem will streamline the detection of mild-to-moderate anxiety and early-stage depression, with a view to flagging individuals at higher risk of developing more debilitating mental health conditions, so their consultations with care professionals can be prioritized. From a sociological perspective, mental health has received significant attention in recent research [20,21]. Studies have focused on improving the understanding of the impact that factors such as age, sex, gender, ethnic background, and socioeconomic status can have on mental ill health, as well as on “the stigmatizing consequences of mental illness as a social status” [22]. It is anticipated that a successful implementation of the proposed mental health and well-being ecosystem will have a beneficial impact on sociological research around mental health. Streamlined ethical sharing of health and well-being data across the ecosystem stakeholders can, in fact, facilitate the identification of factors affecting mental health and well-being across the population and can point to disparities in terms of access to support and care services. More broadly, crowdsourced data collection has been identified as a promising way of facilitating the achievement of the positive impact of the behavioral sciences on policy making [23]. This is based on the observation that behavior often exhibits a high degree of specificity in relation to context, that testing of new solutions in specific settings is required for successful innovation, and that crowdsourced data collection can enable the implementation of financially sustainable and scalable data collection strategies. For these reasons, it is expected that the development of the proposed ecosystem will create ideal conditions for strengthening the positive impact of the behavioral sciences on policy making in relation to mental health and well-being.

The potential of digital technologies in support of mental health and well-being screening is illustrated by the availability of portable electronic devices and mobile software applications and by the data streams that they generate, either passively (using embedded sensors including accelerometers and gyroscopes) or following purposeful user interaction. Large-scale collection, integration, and analysis of data from wearable bio-sensing devices measuring sympathetic nervous system activity and tracking user behavior, combined with analysis of social media data relating to communication patterns and content, have the potential to enable evidence-driven triaging of mental health conditions [24]. This, in turn, can increase the efficiency of support and care services and can facilitate fairer access to resources. Nonetheless, if large-scale adoption of remote screening technology is to be achieved, systemic changes are required within health care organizations, and consistent narratives need to be put in place to facilitate continued engagement of the public, of individuals in need of mental health and well-being support, and of care professionals [25].

Existing mental health screening and diagnostic methods typically rely on in-person one-to-one interaction between individuals in need of mental health support and care professionals. Whereas reliance on telephone and videoconferencing technology for remote consultations can partly address the negative environmental impact associated with exclusive reliance on in-person interaction, it cannot provide a financially sustainable large-scale deployment solution unless combined with efficient triaging [26]. The proposed ecosystem is envisaged to fill this gap, in line with the concept of “Healthcare 5.0,” which encompasses “real-time patient monitoring, ambient control and wellness, and privacy compliance” [27] based on a combination of artificial intelligence, “Internet of Things,” “big data,” as well as high-bandwidth and high-availability networking technologies. It has been suggested that health Information Technologies (ITs) can enable the collection of more objective behavioral health management data and can underpin more effective mental health and well-being screening than traditional approaches relying on questionnaires administered in primary care and educational settings [28]. There have been reports of questionnaire-based methods overestimating socially desirable behaviors, medication adherence being one example, and it has been recognized that the quality of data collected using such questionnaires ultimately rests on accurate recollection of past events by the respondents, which is often limited [28]. Nonetheless, if large-scale mental health and well-being screening strategies enabled by modern digital technologies are to be developed, changes for all stakeholders will be required.

Considering the ethical challenges involved in the adoption of digital interventions for mental health and well-being, policies and frameworks have been developed to address issues of privacy, transparency, and user trust in modern technologies [29-31]. Such issues are of direct relevance to ongoing research aiming to understand barriers to user engagement with digital technologies in support of mental health and well-being [32]. A recent report on attitudes to digital mental health technologies by the Medicines and Healthcare products Regulatory Agency (MHRA) [33] has highlighted the importance of several factors influencing perceptions and attitudes of the public regarding digital innovations in support of mental health and well-being. Such factors include the following: integration within wider support and care services; endorsement from authoritative organizations such as the UK National Health Service, MHRA, and the National Institute for Health and Care Excellence; the inclusion of a human element in addition to digital interaction, with emphasis on empathy; digital technologies and information provenance; arrangements for anonymized data sharing, preferably with public universities and research organizations as opposed to commercial entities; proportionate regulation to minimize the negative impact of scams, commercial advertising, and exploitative attempts at taking advantage of individuals who feel vulnerable; clear terms and conditions; granular user control over the data being shared; clarity regarding responsibilities when problems arise; evidence of feasibility and effectiveness, including success rates and relevant metrics, ideally relying on standardized evaluation frameworks for enhanced transparency. Recent work by the Alan Turing Institute has concentrated on “assurance” as a “process of building trust and justified confidence in a system or technology through engagement and communication” [34], with the aim of developing trustworthy digital mental health care. A framework and methodology were presented, underpinning the definition of principles and guidelines for the development of trustworthy digital innovations. Particular attention was paid to fairness (balanced distribution of risks and benefits across user groups) and to explainability in relation to the use of predictive analytics, considering the importance of such aspects for establishing public trust in innovations enabled by modern technologies.

The concept of “mental health ecosystem” is not new. Recent research has focused on identifying opportunities and challenges for digital innovators with reference to the development of new mobile health tools to promote mental health [9]. The emphasis has been on improving the alignment between the innovation strategies and the perceived relevance and benefits of the innovations to the users. For this reason, it has been recommended that the use of co-design methods, underpinned by insights from behavior theory, be prioritized, with a view to lowering barriers to uptake of the innovations and to fulfilling the user needs in line with users’ values and expectations. Whereas the digital mental health ecosystem proposed in [9] has an emphasis on mental health care provision, the focus of the sociotechnical ecosystem, the design of which is delineated in this article, is on empowering individuals in relation to their own well-being and on enabling large-scale mental health screening and preventative interventions. For this reason, issues relating to screening and health anxiety [35] will need to be considered as part of the design of the ecosystem, in addition to the ethical issues discussed previously.

The following sections present the methods used for identifying the key components of the theoretical foundations for this study, the theoretical framework underpinning the development of the mental health and well-being ecosystem, research aims, working hypotheses, the ecosystem and associated development strategies, and conclusions.

## Methods

### Overview

The methodology adopted for delineating the design of the proposed ecosystem is grounded in critical realism, which identifies 5 key stages in interdisciplinary research: (1) planning, (2) disciplinary reflection, (3) teamwork concentrating on developing understanding across discipline boundaries, (4) knowledge integration, and (5) interdisciplinary understanding of relevant structures and interdependencies [36]. The principle of “epistemological pluralism” [37] has proven particularly useful in the context of this study, in recognition of the existence of different ways of “knowing” and of generating knowledge about complex systems in which components refer to qualitatively different contexts and domains. The mental health and well-being ecosystem discussed in this article is defined via the convergence of technical, semantic, semiotic, social, organizational, and ethical elements, each of which has been a subject of academic and broader professional discourse.

A scoping review of interdisciplinary literature led to the delineation of the proposed design framework. The review included recent developments in relation to semiotics, sense-making, cognitive load, social engagement, health beliefs, health behavior change, corporate social responsibility, diffusion of innovations, explainable artificial intelligence for health care, and ethics in the processing and management of health and well-being data. Google Scholar was used to perform initial searches of scholarly publications across the relevant disciplines, relying on keywords that were iteratively refined based on the results returned. Initial keywords were selected with a view to maximizing the likelihood of retrieving publications relevant to the different aspects of the ecosystem, and included a logical OR of the following: “mental health and wellbeing”; “digital technologies” AND “health and wellbeing”; “artificial intelligence” AND “health and wellbeing”; “data sharing” AND “health and wellbeing”; “health and wellbeing” AND “AI ethics”; “health screening”; “employee wellbeing”; “corporate responsibility” AND “sustainable business models”; “visual ecologies”; “sense-making through metaphors”; “social engagement”; “behavior change” AND “health and wellbeing”; “diffusion of innovations” AND “health and wellbeing”. Where required, the searches were extended to additional databases and digital platforms as part of a process of iterative refinement including consolidation discussions among the authors. Additional databases and platforms included Scopus, ACM Digital Library, Web of Science, and PubMed. Searches were restricted to publications written in English, and articles published within the last 10 years were prioritized. Considering the importance of ethical and regulatory issues in relation to the proposed ecosystem, additional public sources were considered beyond academic publications, most notably MHRA and the Alan Turing Institute regarding the use of software and artificial intelligence as a medical device. The definition of keyword-based queries was refined in the context of scoping and iterative concept consolidation discussions among the authors. The authors’ combined academic and professional expertise spans human-centered technology innovation, artificial intelligence, design for health care innovation, arts therapies, and business transformation. Such a convergence of expert knowledge across academic disciplines and professional practice has enabled the development of the study model, building on established but thus far disjoint theoretical frameworks, as well as the generation of innovative design concepts for the development, implementation, and sustainable deployment of the ecosystem.

### Theoretical Framework

**Figure 1.**
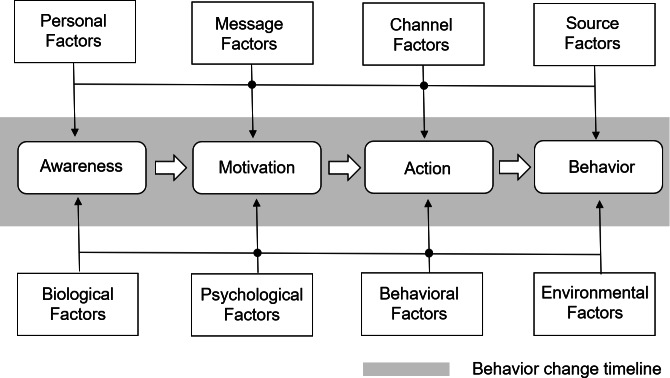
The Integrated-Change Model (adapted from De Vries [40], which is published under Creative Commons Attribution 4.0 International License [45]).

The proposed mental health and well-being ecosystem is meant to create the conditions for a positive change process to take place for individuals by engendering a sense of ownership and agency over health and well-being, and by encouraging the engagement of individuals within communities defined by shared interests and goals. The change process will be facilitated by interactive capabilities accessed via digital and physico-digital interfaces enabling individuals to visualize and intervene in external representations of individual and community health and well-being data. The theoretical framework is the result of the convergence of sustainable business and well-being models documented in the management research literature [38] with behavior change models, especially those that have proven beneficial in modeling mechanisms of change in relation to the adoption of digital health care innovations [39]. The Integrated-Change Model [40], the potential of which for the design of digital health programs has been recently documented [41], is used in the following to elucidate key factors relevant to the ecosystem framework. Figure 1 displays key elements of the Integrated-Change Model (“Awareness,” “Motivation,” “Action,” and “Behavior”) together with factors influencing the change process, including changes in attitudes and behavior with reference to the adoption of digital health innovations. The middle row with boxes within the gray area represents an idealized linear sequence of transitions from “Awareness” to “Motivation,” from “Motivation” to “Action,” and from “Action to Behavior.” The thinner arrows in the figure show which factors influence which of the 4 elements. The filled dots have been included to make it explicit that each factor can affect each of the elements. The directional flows in Figure 1 illustrate the progression and interplay between the key elements of behavior change, namely “Awareness”→“Motivation”→“Action”→“Behavior.” These arrows represent a sequential yet dynamic process. While the primary flow suggests a linear progression, it is important to acknowledge that nonlinear dependencies and feedback loops exist within this model. For instance, the “Awareness” stage serves as the initial step, where individuals recognize the need for change. However, feedback from subsequent stages, such as experiencing barriers during “Action,” can prompt individuals to revisit and deepen their “Awareness.” The “Motivation” stage is influenced not only by the preceding “Awareness,” but also by factors such as support or barriers encountered during “Action” and “Behavior,” which creates a bidirectional relationship. Finally, whereas “Action” leads to tangible efforts toward change, sustained “Behavior” can reinforce “Motivation” or reshape “Awareness” through reflection on outcomes.

**Figure 2.**
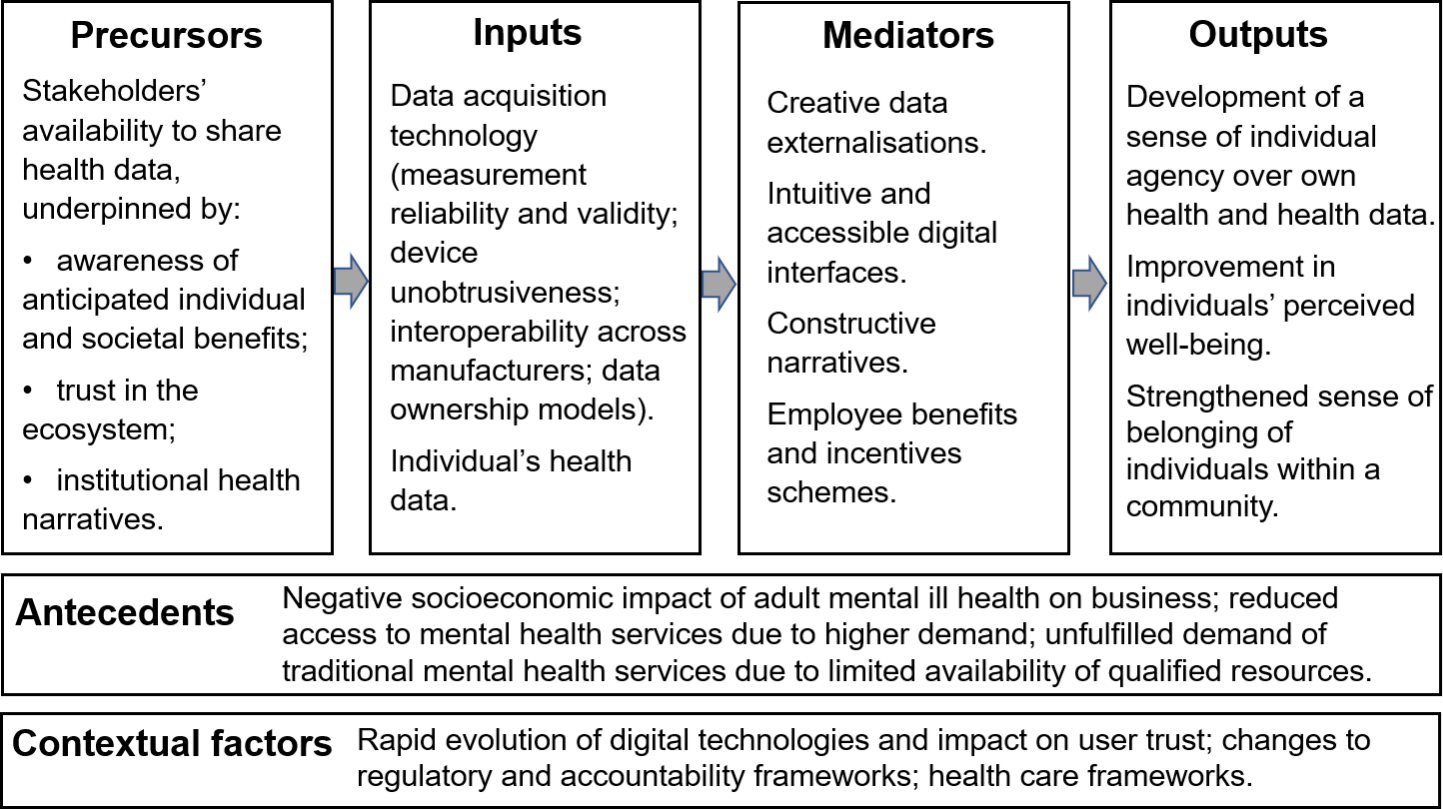
Logic model of the interactive change process to be enabled by the ecosystem.

A logic model of the proposed interactive change process is presented in Figure 2, where precursors are formulated as willingness and availability to share health and well-being data across the ecosystem stakeholders, underpinned by awareness of anticipated benefits and trust in the ecosystem. For this discussion, “trust” is defined as epistemic confidence, identity centrality, or a combination of the two. Epistemic confidence is the “degree to which someone feels a belief state approximates [...] clear contents, objective truth, and rational justification” [42], whereas identity centrality is the “degree to which someone experiences a belief state as part of their social identity” [42], where “social identity” is defined as “a cluster of psychological states and behavioral dispositions that constitute someone as a member of an actual or potential in-group, or that an individual uses to achieve a desired social position” [42]. The definition and relevance to the ecosystem of the concept of “trust,” which plays a critical role in the framework as reflected in Figure 2, is grounded in recent developments [40,42-44].

Considering the different stakeholders involved in the ecosystem, it is expected that the role played by epistemic confidence and identity centrality in the context of the interactive change process will depend on context. “Antecedents” and “Contextual Factors” have been separated from “Precursors” in Figure 2, to clarify that the latter are prerequisites to the use of individuals’ health and well-being data as input to the interactive change process within the ecosystem. In addition to health and well-being data externalizations augmented by accessible interactive capabilities, mediators of positive change include constructive and consistent narratives around mental health as well as employee benefits and incentive schemes. Recent research has highlighted a need for additional depth to the ongoing discourse across academics and practitioners, including HR professionals, with a view to identifying how employee benefits and incentives schemes can best be devised and embedded within existing corporate social responsibility programs [46]. Care should be taken to mitigate risks associated with unintended effects, including potential negative impacts on employee mental health. The convergence of such future studies with existing efforts to establish scientific tools for the assessment of employee psychological, social, subjective, and workplace-related well-being [47] holds promise to feed into the development of the proposed mental health and well-being ecosystem.

Table 1 summarizes key features of the interfaces providing access to the health and well-being data externalizations, mapped to behavior change stages (“Awareness,” “Motivation,” “Action,” and “Behavior”), with reference to the interactive change process as presented in the Integrated-Change Model (Figure 1). Individual characteristics relevant to the different behavior change stages have been included in the table. Attitudes are generally determined by individual and contextual factors such as individual predisposition to the use of digital technologies and peer influence. Similarly, intentions can be affected by individuals’ attitudes and peer influence. Table 2 reports a list of factors from the Integrated-Change Model affecting individuals’ willingness and abilities to engage with different features of the proposed physico-digital interfaces.

**Table 1.** Features of the interfaces to health and well-being data externalizations, mapped to behavior change stages and to relevant individual characteristics.

Behavior change stage and individual characteristics	Interface feature
Awareness	
Cognizance	Signposting of information and advice
Knowledge	Signposting of information and advice
Risk perception	Signposting of information and advice
Perception of interactive cues	Inclusive access to interactive functionalities
Motivation	
Attitude	—^a^
Social influences	Capability of interacting with individuals and groups
Self-efficacy	Inclusive access to interactive functionalities
Intention	—
Action	
Willingness to overcome barriers	Signposting of information and advice
Willingness to plan	Capability of interacting with individuals and groups
Willingness to enact	Capability of interacting with individuals and groups
Behavior	
Interest in own well-being data	Functionalities for individuals to track changes to their own well-being and behavior over time
Interest in community well-being data	Functionalities for individuals to track changes to data originating from multiple users over time

a Not applicable.

**Table 2.** Factors influencing individuals’ willingness and abilities to engage with the proposed interfaces to creative externalizations of health and well-being data, extracted from the Integrated-Change Model.

Factor	Interface feature
Biological	Design to enhance accessibility
Psychological	Design to enhance accessibility
Behavioral	Design to enhance accessibility
Environmental	Design to enhance accessibility
Personal	Signposting of information and advice
Message	Capability of interacting with individuals and groups, with an option for anonymity
Channel	Interactive access to data with varying degree of granularity
Source	Reliance on authoritative channels for signposting of information and advice

In the context of the Integrated-Change Model, the “Action” stage encompasses the processes and capacities required to move from motivation to pragmatic behaviors. The inclusion of “Willingness to overcome barriers” with reference to this stage reflects the active effort that individuals must make to address obstacles that arise when planning or enacting behaviors. Barriers, in this sense, are seen not as static, pre-existing factors but as dynamic challenges that individuals encounter and address during the action-taking process. For example, accessing appropriate resources or engaging with others to resolve these barriers may depend on an individual’s willingness and capability to engage actively. Similarly, “Capability of interacting with individuals and groups” is categorized under “Action” because it directly influences the ability to execute planned behaviors in a social context. Whereas “capability” might traditionally be seen as a preceding factor (or skill), it is treated as part of the “Action” process in the context of this framework because the application of capability is what enables individuals to translate plans into real-world behaviors. This categorization emphasizes the dynamic and iterative nature of actions, particularly in collaborative and group-based contexts, integral to taking meaningful steps toward change.

**Figure 3.**
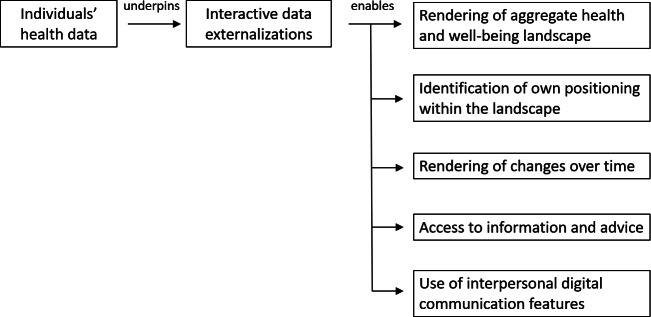
Schematic representation of how individuals’ health and well-being data will underpin key functionalities enabled by interactive data externalizations.

A schematic representation of functional relationships between individuals’ health and well-being data, data externalizations, and features of the interfaces is provided in Figure 3, with emphasis on functionalities to facilitate access to information and advice and to encourage the engagement of individuals within communities.

The factors listed in Table 2 are the same as reported in [40] as “Preceding Factors” and “Information Factors”: “Personal Factors,” “Message Factors,” “Channel Factors,” “Source Factors,” “Biological Factors,” “Psychological Factors,” “Behavioral Factors,” and “Environmental Factors.” “Message Factors,” “Channel Factors,” and “Source Factors” are information-specific and refer to communication between individuals and across communities. Examples of relevant factors with reference to the mental health and well-being ecosystem and the associated physico-digital interfaces to the proposed creative externalizations of health and well-being data include the following: individuals’ circumstances affecting engagement within communities, for example, limited time due to work or family commitments (“Personal Factors”); the content of communication between individuals (“Message Factors”); the communication channels used, for example, face to face as opposed to the use of digital technologies between individuals, and Web interfaces regarding individuals accessing health information and guidance (“Channel Factors”); information provenance, for example, authoritative organizations in relation to the provision of health and well-being advice (“Source Factors”); individuals’ health as a result of genetic predisposition to specific conditions (“Biological Factors”); individuals’ self-efficacy in relation to management of own well-being (“Psychological Factors”); individuals’ degree of engagement within local communities (“Behavioral Factors”); availability of local initiatives promoting individuals’ engagement within communities (“Environmental Factors”).

### Research Aims

The conceptual modeling presented in this article aims to promote individuals’ engagement with health and well-being by enhancing awareness and fostering a sense of agency over personal well-being while encouraging community participation. Design of creative externalizations of live multimodal health and well-being data streams is envisaged, together with the definition of dedicated engagement strategies to engender a sense of ownership of personal health data. Further engagement of individuals within local communities is expected to lead to benefits for both individuals and communities and to underpin the sustainable operation of the ecosystem via continued engagement of individuals in the proposed ethical health and well-being data-sharing program. Nonetheless, strategies for promoting engagement should also consider the involvement of community leaders and influencers, including organizations representing minority groups.

Business organizations will be empowered with scalable ethical methods of monitoring employee well-being and tailoring interventions toward increased productivity and improved workforce resilience. Gaps have been identified in terms of ethical and regulatory frameworks to cover collection, storage, exchange, and processing of individuals’ health data (including the use of inferential algorithms for screening purposes) with reference to smart health care applications, particularly in the context of prospective real-time patient monitoring and management systems enabled by emerging technologies [27]. For this reason, addressing regulatory gaps in data governance is a key aim in relation to the development of the ecosystem, along with identifying effective health and well-being data sources and integration strategies. Finally, the research seeks to streamline the ethical exchange of health and well-being data across organizations and to develop metrics and algorithms for monitoring health and well-being changes over time, thereby enhancing the integration and effectiveness of care services. The research aims are summarized below.

- A1 (Promote individuals’ engagement, community collaboration, and community empowerment): To increase individuals’ awareness of (and contribute to engendering a sense of agency over) personal well-being, at the same time promoting the engagement of individuals within their communities. To engage with organizations representing local communities, including minority groups, with a view to encouraging further the promotion of the ecosystem.
- A2 (Design externalizations of mental health and well-being data): To identify relatable health and well-being data experiential strategies to enable meaningful and sustained engagement of individuals, to facilitate an objective assessment of personal and community mental health, and to streamline access to peer and specialist support.
- A3 (Empower businesses): To provide business organizations with ethical, cost-effective, and scalable methods of quantifying and monitoring employee well-being, and tailoring interventions to increase employee well-being and business productivity.
- A4 (Address regulatory gaps): To address current regulatory gaps regarding the governance of individuals’ and organizational data as well as data exchange between care providers and business organizations, thereby identifying ways of implementing changes to existing policies and regulations.
- A5 (Select and integrate health and well-being data sources): To identify what streams of individuals’ health and well-being data (including data from bio-sensors, mobile software applications, and self-reports) and what data integration strategies are best suited for maximizing benefits to individuals, communities, business organizations, and care service providers.
- A6 (Streamline exchange of health and well-being data): To facilitate the responsible exchange of individuals’ health and well-being data within integrated care services as well as between care services and business organizations, with a view to supporting the operational integration of care and support services.
- A7 (Develop algorithms for monitoring health and well-being changes): To generate equitable metrics (and algorithms relying on them) to enable tracking of health and well-being changes over time at individual and community levels.

### Working Hypotheses

#### Overview

The working hypotheses underlying the design and development of the ecosystem are discussed in this section with reference to relevant theories and models.

#### Overarching Working Hypothesis

A health and well-being data-sharing program can be designed for equitability and trust, to underpin a sociotechnical ecosystem comprising individuals, care providers, and business organizations, enabling ethical screening and promotion of mental health and well-being for the benefit of individuals and communities.

Considering the complexity of the proposed ecosystem and the interdisciplinary character of the research, the overarching working hypothesis is best broken down into multiple hypotheses relevant to different aspects of the ecosystem and to different beneficiaries of the innovation. A list of working hypotheses and relevant theoretical and methodological underpinnings is provided below.

#### H1: Empowerment of Individuals, Community Collaboration, and Community Empowerment

Individuals can benefit from awareness and engagement with their health and well-being data as well as from engagement with local communities in terms of improved well-being. Community leaders and influencers can be involved in programs to support the sustainable operation of the ecosystem. Theories, models, and methods: sense-making theory [48] and theory of social engagement [49].

#### H2: Employee Well-Being

Access to aggregate anonymized workforce health and well-being data can enable businesses to develop targeted interventions to improve employee well-being and productivity. Theories, models, and methods: corporate social responsibility [50].

#### H3: Health Data and Preventive Care

Large-scale collection and integration of personal health data can support national health care services and integrated care providers in implementing preventive and early-stage interventions. The primary aim is to reduce costs associated with the diagnosis and treatment of severe and chronic mental health conditions. Theories, models, and methods: diffusion of innovations [51,52].

#### H4: Sustained Participation

Creative and aesthetic externalizations of health and well-being data, augmented with advanced physico-digital interactivity and combined with benefits and incentive structures, can foster active and sustained participation in the data-sharing program. Theories, models, and methods: sense-making theory [48]; semiotic models [53]; cognitive load theory [54]; and affordance theory, recently reviewed in relation to information systems [55].

#### H5: Data Integration

Collection, integrity assessment, and integration of individuals’ health and well-being data can be scaled up to local, regional, and national levels. Theories, models, and methods: diffusion of innovations [51,52] and corporate social responsibility [50].

#### H6: Actionable Insights

Actionable insights can be generated from individuals’ health and well-being data with different degrees of granularity, to monitor and promote the health and well-being of individuals and communities. Theories, models, and methods: diffusion of innovations [51,52], explainable artificial intelligence for health care [27], and validation methods for machine learning models in medicine [56].

#### H7: Monitoring of Health and Well-Being Changes

Modern predictive analytics techniques can underpin real-time monitoring of health and well-being changes at individual, community, and societal levels, based on quantitative metrics suitable for streamlining equitability assessment. Theories, models, and methods: diffusion of innovations [51,52], explainable artificial intelligence for health care [27], validation methods for machine learning models in medicine [56], and contributing to the ongoing program for change to the UK regulatory framework for software and artificial intelligence as a medical device [57].

### The Mental Health and Well-Being Ecosystem and Strategies for Its Development

#### The Mental Health and Well-Being Ecosystem

**Figure 4.**
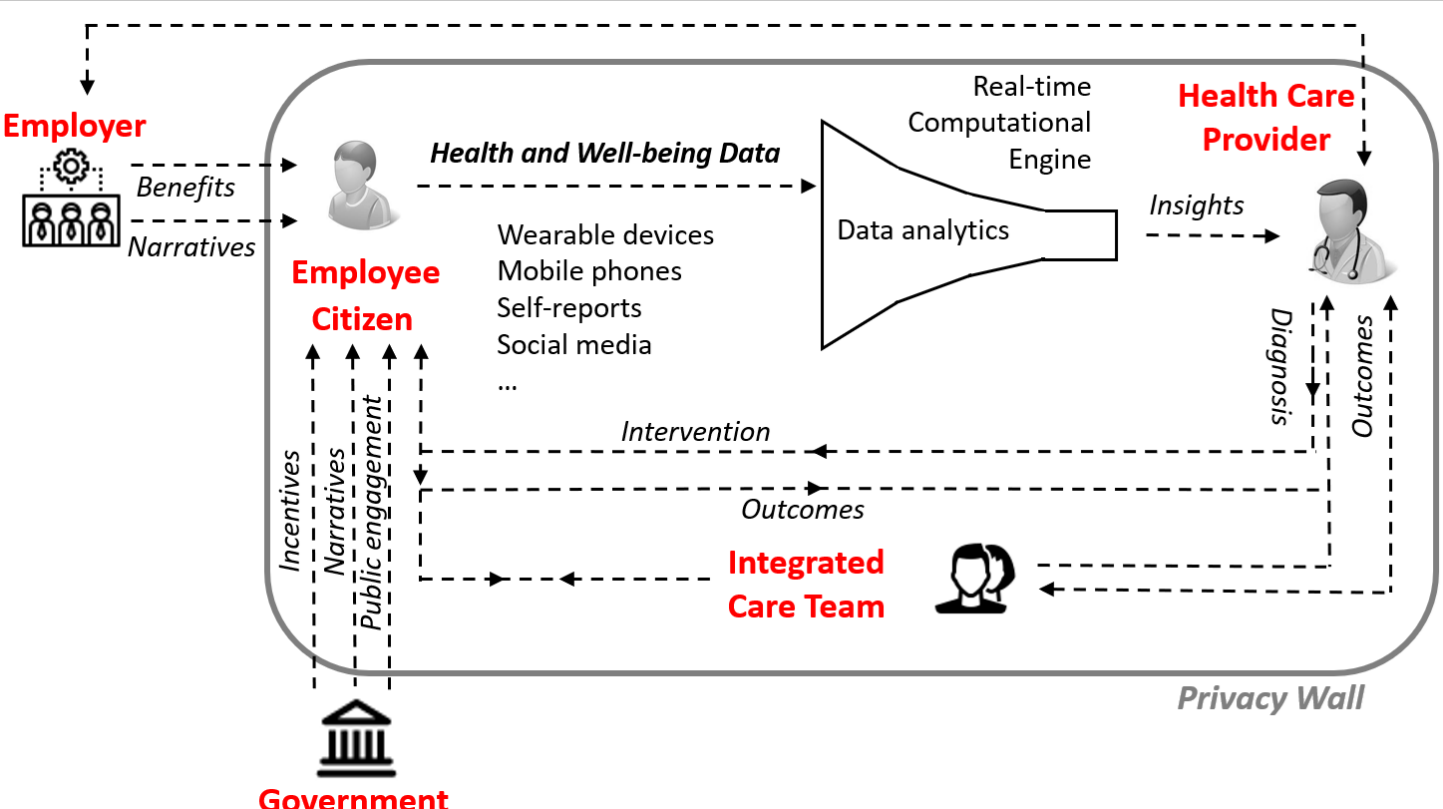
Schematic representation of the proposed sociotechnical ecosystem.

A schematic representation of the proposed ecosystem is provided in Figure 4, where roles are presented for individuals; business organizations in their capacity as employers, health care providers, and integrated care teams; and government authorities. Integrated care teams comprise professionals with complementary knowledge, skills, and experience—they often include Nurses, Occupational Therapists, and Psychology specialists working alongside Social Workers, Physiotherapists, physical health Occupational Therapists, and General Practitioners. Arrows in the figure reflect the flow of information or causal relations**,** as relevant.

The operation of the system will rely on 3 pillars, as outlined below.

- Pillar 1*:* An employee engagement program, including the provision of suitable incentives and benefits schemes.
- Pillar 2: A public engagement program to favor engagement of individuals in digitally enhanced mental health and well-being screening.
- Pillar 3: A scalable IT infrastructure to enable the collection and integration of data across a range of sources including portable electronic devices, smartphone software applications, social media communication, and self-reports.

Table 3 provides additional detail about development stages and relevant stakeholders with reference to the development of the 3 pillars. The adoption of an interdisciplinary approach is a key requirement for the successful development of this research program. Development priorities in relation to the 3 pillars are outlined below. It is recommended that designers and design researchers be involved across the 3 pillars at the relevant development stages. This will create favorable conditions for the consolidation of the conceptualization program and for the innovations to be aligned with user values, needs, and requirements as well as with the expectations of the different stakeholders.

**Table 3.** Development stages and relevant stakeholders with reference to the 3 pillars of the mental health and well-being ecosystem.

Pillar	Stakeholders	Development stage
Pillar 1**:** Engagement, incentives, and benefits schemes	Designers and design researchers; employers; government; health care staff	Program implementation
Pillar 2: Constructive and consistent mental health narratives	Designers and design researchers; behavior change specialists; health care staff; government organizations	Program conceptualization
Pillar 3: Scalable IT infrastructure integrated with health care systems	Designers and design researchers; technology developers Designers and design researchers; AI researchers and data scientists; health care systems; government authorities; policy makers	Technical development Program implementation

#### Pillar 1: Engagement, Incentives, and Benefits Schemes

Identifying operational mechanisms to encourage continued engagement of individuals with the ecosystem and with its data-sharing program will play an important role in the development process. The role of strategies incorporating multifaceted sense-making through metaphors [48] has been investigated in the domain of well-being data visualization, and successful health and education research has been conducted relying on metaphors based on visual ecologies [58,59]. Visual metaphors representing social ecosystems can offer scope for expressing “dynamic landscapes” of live metrics, for example, relying on symbolic biospheres, in such a way that position and movement of individuals’ health and well-being data representations within the landscape can symbolize significant personal well-being milestones and challenges, informed by health and social paradigms and based on personal goals. Ways of incorporating population-level metrics within the interactive externalizations of individual health and well-being data will be investigated during the development of the ecosystem, so that personal data can be contextualized within continuously evolving geographic community representations. This component of the ecosystem ultimately builds on the hypothesis that simple contextualization of health and well-being data within a collective “landscape” can engender a sense of social connectedness and ultimately improve individual and community mental health. Work to develop this pillar will include the definition of suitable employee incentives and benefits schemes, as well as the identification of methods of integrating employee health and well-being assessment workflows within appraisal and annual review processes. It is expected that the involvement of designers and design researchers, starting with the pillar 1 conceptualization stage, will facilitate the employment of generative research methods and co-design practices and will create ideal conditions for the user needs to be accurately identified and translated into actionable system requirements.

#### Pillar 2: Constructive and Consistent Mental Health Narratives

The promotion (by government authorities, care providers, and business organizations) of constructive and consistent mental health narratives for public consumption will be important for addressing issues of stigmatization that can negatively impact individuals’ perceptions and attitudes toward the ecosystem. The narratives should be devised as public institutional messages, with the dual aim of encouraging individuals’ stronger agency over their own well-being and of inviting the development of additional interventions by business organizations to foster employees’ health and well-being in addition to performance assessment. By embedding such narratives into every aspect of the ecosystem, from design to policy advocacy, the research program will foster a holistic, inclusive, and empowering approach to health and well-being for individuals and communities. Communication strategies implemented by national governments and employers should therefore be devised with the active participation of public, patient, clinical, health care management, and third-sector stakeholders. Examples of narratives to be considered include those focusing on modern integrated views of well-being along mental, social, and physical dimensions [60], as opposed to others with more explicit clinical connotations—for example, with reference to the diagnosis of anxiety, depression, and other mental health conditions. It is recommended that designers and design researchers be involved at the development stage, so their expertise can feed into the definition and implementation of iterative testing and design refinement strategies. The emphasis should be on ecosystem tools and interfaces, with the aim of enhancing accessibility and inclusivity and facilitating collaboration between end users and technical teams.

#### Pillar 3: Scalable IT Infrastructure Integrated With Health Care Systems

The operation of the ecosystem is to be underpinned by an IT infrastructure and suitable digital platforms, including features to enable secure data storage, data processing, and generation of actionable insights. The infrastructure and the digital platforms should be developed for scalability and cost-effectiveness in coordination with user groups and stakeholders; the emphasis should be on addressing both systemic and infrastructural barriers to deployment, including those associated with limited interoperability across hardware and software providers. The benefits of data-driven health and well-being insights for individuals, care professionals, caregivers, business managers, and health care management professionals will be maximized thanks to the adoption of digital interfaces designed for interpretability and ease of use, to replace existing dashboards that often require a degree of technical competency for efficient use. Dedicated interfaces will streamline access to health and well-being data with different degrees of granularity for research and government organizations, for example, to generate epidemiology and public health insights in support of policy making. The ability to quantify, track over time, and display informative visual representations (based on interpretable metaphors) of health and well-being changes at individual and community levels—potentially, also at the level of individual support and care services—is expected to facilitate the task of making sense of health and well-being data, often complex in nature. Approaches have been proposed for secure storage and analysis of individuals’ health data relying on federated machine learning techniques and associated information systems architectures. Such approaches typically rely on the storage and processing of data within local computational nodes, combined with the shared availability of machine learning models built using data aggregated from across the system [61]. These methods and techniques hold the potential for addressing privacy concerns while at the same time enabling the generation of a large-scale evidence base. At the implementation stage, designers and design researchers are expected to play an essential role in evaluating the success of the system in real-world scenarios, relying on data-driven insights for further refining tools and interfaces. Their continued involvement will support the adaptability and long-term sustainability of the ecosystem.

Both the data externalizations and the associated interactive features are to be designed with the involvement of the public and patients, and in coordination with key stakeholders including managers and health care staff. Attention will have to be paid to suitable degrees of granularity for access to health and well-being data by individuals and organizations, as well as to the circumstances where such access is appropriate. Similarly, the possibility of negative affect states being transferred across members of a community [62,63] via individuals’ interactions with the data externalizations will have to be considered when designing the interactive features.

The availability of aggregate anonymized data about employee health and well-being will facilitate the employers’ task of implementing tailored support strategies. Access to such data holds additional potential for helping job applicants in selecting the most suitable prospective employers, for example, based on statistics about the average degree of mental health and well-being corresponding to different business locations. Care service providers are expected to benefit from the ecosystem in terms of more efficient management of early signs of individuals’ psychological distress, before conditions exacerbate and require more sustained reliance on care services. Finally, the practice of ethically sharing individuals’ health and well-being data that fulfill reliability and validity requirements is anticipated to streamline the exchange of information and insights across integrated care teams consisting of multiple service providers. This, in turn, will contribute to more efficient delivery of—and more inclusive access to—support and care services.

An initial pilot study should be conducted to understand opportunities and barriers to engaging the public in mental health and well-being screening interventions relying on ethical sharing of individuals’ health and well-being data across the ecosystem stakeholders. The involvement of diverse groups of individuals in a human-centered design program is encouraged. This will create favorable conditions for promoting engagement with the ecosystem across the population, with emphasis on underrepresented and underserved communities. Attention should be paid to minority ethnic groups, members of the LGBTQ+ (Lesbian, gay, bisexual, transgender, queer or questioning, intersex, asexual, and others) community, and individuals residing in disadvantaged geographical areas or otherwise less likely to have access to mental health screening services due to socioeconomic status**,** age, or sex. Further development should focus on the role to be played by business organizations and care providers within the ecosystem, on regulatory and policy-making implications, and on developing the underpinning technological infrastructure.

## Conclusions

This concept paper has introduced a comprehensive sociotechnical ecosystem framework to enable ethical mental health and well-being screening on a large scale, with a view to promoting health and well-being for individuals and communities. The convergence of individuals, care providers, and business organizations, supported by government authorities and building on a shared willingness to contribute personal and institutional health and well-being data, has been proposed as a key enabler of the ecosystem, with anticipated benefits in terms of streamlined ethical collection, integration, and sharing of health and well-being data. Reliance on predictive numerical models will enable the generation of actionable insights to be shared across stakeholders with different degrees of granularity, with anticipated benefits for individuals, communities, care service providers, and business organizations. It is also anticipated that streamlined ethical health and well-being data exchange across the ecosystem stakeholders will benefit sociological discourse around mental health by providing an integrated knowledge base. Such a knowledge base will support the identification of inequities across the population in terms of mental health and well-being outcomes as well as access to support and care services. Whereas the focus of this study is on the UK health service provision, the generalizability of the proposed ecosystem framework is underlined by the recommended engagement of all stakeholders throughout the design process: this underpins the adaptability of the framework to different economic, regulatory, and cultural environments. By integrating ecological design principles, the framework emphasizes the importance of iterative engagement with local stakeholders within complex human-technological environments. The proposed engagement is to include policy makers, community leaders, and end users, taking into consideration the characteristics of the interactive digital and physical environment. The goal is to maximize the alignment of the ecosystem components with the specific needs and values of the user population. For instance, in low-resource settings, adaptations could focus on leveraging cost-effective digital tools and on prioritizing interventions that address systemic barriers such as limited health care access. On the other hand, in high-resource settings, the framework might emphasize advanced analytics and personalized interventions while adhering to the relevant regulatory requirements. Additionally, cultural adaptations can rely on the embedding of local norms, languages, and beliefs into the design and delivery of digital tools to foster trust and engagement. Tailoring implementation strategies to varying contexts will enhance the generalizability of the framework and its anticipated positive impact in terms of improved global mental health outcomes.

The theoretical underpinning of the ecosystem is the result of the coalescence of existing well-being and sustainable business frameworks, combined with health behavior change models. Equally, the framework relies on the systemic integration of disjoint services and information sources, enhanced using predictive analytics and underpinned by a robust data governance framework. Working hypotheses have been presented, with emphasis on the engagement of individuals with health and well-being data, facilitated by creative externalizations of multimodal live data streams augmented with advanced physico-digital experiences, to enhance individual and collective engagement as well as agency rooted in shared interests and goals. Proactive involvement of all stakeholders from an early stage has been proposed with the aim of nurturing a conducive environment that will maximize the public, organizational, and health care benefits of the ecosystem. A research agenda based on the ecosystem framework has been proposed to enhance transdisciplinary socially inclusive discourse. Addressing ethical considerations in the development of complex health systems, the governance of personal health data, and policy implications will be key to advancing mental health and well-being screening to the highest ethical standards, with a view to maximizing positive societal impact.

## Abbreviations

IT: Information Technology
LGBTQ+: Lesbian, gay, bisexual, transgender, queer or questioning, intersex, asexual, and others
MHRA: Medicines and Healthcare products Regulatory Agency

## References

[1] Knapp M, McDaid D, Parsonage M (2011). Mental health promotion and mental illness prevention: the economic case. GOVUK.

[2] Mental Health Foundation.

[3] NHS England (2023). https://www.england.nhs.uk/long-read/delivery-plan-for-recovering-access-to-primary-care-2.

[4] Pomerantz A, Cole BH, Watts BV, Weeks WB (2008). Improving efficiency and access to mental health care: combining integrated care and advanced access. Gen Hosp Psychiatry.

[5] Ataguba G, Orji R (2024). Toward the design of persuasive systems for a healthy workplace: a real-time posture detection. Front Big Data.

[6] Arakawa Y (2019). Sensing and changing human behavior for workplace wellness. J Inf Process.

[7] Forbes Öste H (2016). BE-ING @WORK: wearables and presence of mind in the workplace. proquest number: 10009357. PhD dissertation.

[8] Mettler T, Wulf J (2019). Physiolytics at the workplace: affordances and constraints of wearables use from an employee’s perspective. Inf Syst J.

[9] Spadaro B, Martin-Key NA, Bahn S (2021). Building the digital mental health ecosystem: opportunities and challenges for mobile health innovators. J Med Internet Res.

[10] Vial S, Boudhraâ S, Dumont M (2022). Human-centered design approaches in digital mental health interventions: exploratory mapping review. JMIR Ment Health.

[11] Corrigan PW, Druss BG, Perlick DA (2014). The impact of mental illness stigma on seeking and participating in mental health care. Psychol Sci Public Interest.

[12] Evans-Lacko S, Koeser L, Knapp M, Longhitano C, Zohar J, Kuhn K (2016). Evaluating the economic impact of screening and treatment for depression in the workplace. Eur Neuropsychopharmacol.

[13] Silverman BG, Hanrahan N, Bharathy G, Gordon K, Johnson D (2015). A systems approach to healthcare: agent-based modeling, community mental health, and population well-being. Artif Intell Med.

[14] McGill E, Er V, Penney T (2021). Evaluation of public health interventions from a complex systems perspective: a research methods review. Soc Sci Med.

[15] Lawrence A, Weber J, Hill VD, Wasieleski DM (2017). Business and Society: Stakeholders, Ethics, Public Policy.

[16] Stawarz K, Preist C, Coyle D (2019). Use of smartphone apps, social media, and web-based resources to support mental health and well-being: online survey. JMIR Ment Health.

[17] Burr C, Taddeo M, Floridi L (2020). The ethics of digital well-being: a thematic review. Sci Eng Ethics.

[18] Shah SGS, Nogueras D, van Woerden HC, Kiparoglou V (2020). The COVID-19 pandemic: a pandemic of lockdown loneliness and the role of digital technology. J Med Internet Res.

[19] Sequeiros H, Oliveira T, Thomas MA (2022). The impact of IoT smart home services on psychological well-being. Inf Syst Front.

[20] Major B, O’Brien LT (2005). The social psychology of stigma. Annu Rev Psychol.

[21] Link BG, Phelan JC, Johnson R, Turner R, Link B (2014). Sociology of Mental Health’ SpringerBriefs in Sociology.

[22] Markowitz F (2005). Sociological Models of Mental Illness Stigma.

[23] Kravitz DJ, Mitroff SR, Callahan-Flintoft C, Oie KS (2024). Crowdsourced data collection opens new avenues for the behavioral sciences to impact real-world applications. Policy Insights Behav Brain Sci.

[24] Sheikh M, Qassem M, Kyriacou PA (2021). Wearable, environmental, and smartphone-based passive sensing for mental health monitoring. Front Digit Health.

[25] Wherton J, Greenhalgh T, Hughes G, Shaw SE (2022). The role of information infrastructures in scaling up video consultations during COVID-19: mixed methods case study into opportunity, disruption, and exposure. J Med Internet Res.

[26] Chew AMK, Ong R, Lei HH (2020). Digital health solutions for mental health disorders during COVID-19. Front Psychiatry.

[27] Saraswat D, Bhattacharya P, Verma A (2022). Explainable AI for healthcare 5.0: opportunities and challenges. IEEE Access.

[28] Haberer JE, Trabin T, Klinkman M (2013). Furthering the reliable and valid measurement of mental health screening, diagnoses, treatment and outcomes through health information technology. Gen Hosp Psychiatry.

[29] Smith KA, Blease C, Faurholt-Jepsen M (2023). Digital mental health: challenges and next steps. BMJ Ment Health.

[30] Vayena E, Haeusermann T, Adjekum A, Blasimme A (2018). Digital health: meeting the ethical and policy challenges. Swiss Med Wkly.

[31] Wies B, Landers C, Ienca M (2021). Digital mental health for young people: a scoping review of ethical promises and challenges. Front Digit Health.

[32] Borghouts J, Eikey E, Mark G (2021). Barriers to and facilitators of user engagement with digital mental health interventions: systematic review. J Med Internet Res.

[33] Humphreys J, Gill M, Rooney S, Ahmad Z (2024). Digital mental health technology: user and public perspectives -- research report from woodnewton. MHRA Medicines and Healthcare Products Regulatory Agency (MHRA).

[34] (2023). Trustworthy and ethical assurance of digital health and healthcare. The Alan Turing Institute.

[35] Barbek RME, Makowski AC, von dem Knesebeck O (2022). Social inequalities in health anxiety: a systematic review and meta-analysis. J Psychosom Res.

[36] Danermark B (2019). Applied interdisciplinary research: a critical realist perspective. J Crit Realism.

[37] Miller TR, Baird TD, Littlefield CM, Kofinas G, Chapin III FS, Redman CL (2008). Epistemological pluralism: reorganizing interdisciplinary research. Ecol Soc.

[38] Lin G, Wei W, Zhu W (2015). The Principle of Profit Models.

[39] Hoare A (2019). Aspects of Digital Change.

[40] de Vries H (2017). An integrated approach for understanding health behavior; the I-change model as an example. PBSIJ.

[41] Cheung KL, Hors-Fraile S, Vries H, Syed-Abdul S, Zhu X, Fernandez-Luque L (2021). Digital Health - Mobile and Wearable Devices for Participatory Health Applications.

[42] Van Leeuwen N (2022). Two concepts of belief strength: epistemic confidence and identity centrality. Front Psychol.

[43] Burr C, Leslie D (2023). Ethical assurance: a practical approach to the responsible design, development, and deployment of data-driven technologies. AI Ethics.

[44] Tajfel H, Turner JC, Jost JT, Sidanius J (2004). Political Psychology.

[45] Attribution 40 international (CC BY 40). Creative Commons.

[46] Dahl MS, Pierce L (2020). Pay-for-performance and employee mental health: large sample evidence using employee prescription drug usage. AMD.

[47] Pradhan RK, Hati L (2022). The measurement of employee well-being: development and validation of a scale. Glob Bus Rev.

[48] Turner JR, Allen J, Hawamdeh S, Mastanamma G (2023). The multifaceted sensemaking theory: a systematic literature review and content analysis on sensemaking. Systems.

[49] Johnston K, Johnston K, Taylor M (2018). The Handbook of Communication Engagement (Handbooks in Communication and Media).

[50] Lindgreen A, Swaen V (2010). Corporate social responsibility. Int J Management Reviews.

[51] Rogers EM (2003). Diffusion of Innovations.

[52] Dearing JW, Cox JG (2018). Diffusion of innovations theory, principles, and practice. Health Aff (Millwood).

[53] Cobley P, Schulz PJ (2013). Handbooks of Communication Science.

[54] Kirschner PA, Sweller J, Kirschner F, Zambrano R. J (2018). From cognitive load theory to collaborative cognitive load theory. Intern J Comput-Support Collab Learn.

[55] Wang H, Wang J, Tang Q (2018). A review of application of affordance theory in information systems. JSSM.

[56] Cabitza F, Campagner A, Soares F (2021). The importance of being external. methodological insights for the external validation of machine learning models in medicine. Comput Methods Programs Biomed.

[57] MHRA (2023). Software and AI as a medical device change programme roadmap. GOVUK.

[58] Havsteen-Franklin D, de Knoop J, Agtarap T, Hackett S, Haeyen S (2023). Evaluation of an arts therapies approach to team development for non-acute healthcare teams in low control and high-pressure environments. Arts Psychother.

[59] Havsteen-Franklin D, Cooper J, Anas S (2023). Developing a logic model to support creative education and wellbeing in higher education. Cogent Education.

[60] Gothe NP, Ehlers DK, Salerno EA, Fanning J, Kramer AF, McAuley E (2020). Physical activity, sleep and quality of life in older adults: influence of physical, mental and social well-being. Behav Sleep Med.

[61] Rehman A, Abbas S, Khan MA, Ghazal TM, Adnan KM, Mosavi A (2022). A secure healthcare 5.0 system based on blockchain technology entangled with federated learning technique. Comput Biol Med.

[62] Paukert AL, Pettit JW, Amacker A (2008). The role of interdependence and perceived similarity in depressed affect contagion. Behav Ther.

[63] Barsade SG, Coutifaris CGV, Pillemer J (2018). Emotional contagion in organizational life. Res Organ Behav.

